# Accumulation mode particles and LPS exposure induce TLR-4 dependent and independent inflammatory responses in the lung

**DOI:** 10.1186/s12931-017-0701-z

**Published:** 2018-01-22

**Authors:** Angela M. Fonceca, Graeme R. Zosky, Elizabeth M. Bozanich, Erika N. Sutanto, Anthony Kicic, Paul S. McNamara, Darryl A. Knight, Peter D. Sly, Debra J. Turner, Stephen M. Stick

**Affiliations:** 1 School of Paediatrics and Child Health, University of Western Australia, Nedlands, WA Australia; 2 Telethon Kids Institute, Subiaco, WA Australia; 3 Department of Respiratory Medicine Princess Margaret Hospital for Children Perth, Subiaco, WA Australia; 4 Centre for Cell Therapy and Regenerative Medicine, School of Medicine and Pharmacology, The University of Western Australia, Nedlands, WA 6009 Australia; 5 Department of Women’s and Children’s Health, Institute of Translational Medicine, University of Liverpool, Liverpool, UK; 6 School of Biomedical Sciences and Pharmacy, University of Newcastle, Callaghan, NSW Australia; 7 Priority Research Centre for Asthma and Respiratory Disease, Hunter Medical Research Institute, Newcastle, NSW Australia; 8 Department of Anesthesiology, Pharmacology and Therapeutics, University of British Columbia, Vancouver, Canada; 9 Queensland Children’s Medical Research Institute, University of Queensland, Royal Children’s Hospital, Herston, QLD Australia

**Keywords:** Asthma, TLR-4, PM, LPS, AMP, COPD

## Abstract

**Background:**

Accumulation mode particles (AMP) are formed from engine combustion and make up the inhalable vapour cloud of ambient particulate matter pollution. Their small size facilitates dispersal and subsequent exposure far from their original source, as well as the ability to penetrate alveolar spaces and capillary walls of the lung when inhaled. A significant immuno-stimulatory component of AMP is lipopolysaccharide (LPS), a product of Gram negative bacteria breakdown. As LPS is implicated in the onset and exacerbation of asthma, the presence or absence of LPS in ambient particulate matter (PM) may explain the onset of asthmatic exacerbations to PM exposure.

This study aimed to delineate the effects of LPS and AMP on airway inflammation, and potential contribution to airways disease by measuring airway inflammatory responses induced via activation of the LPS cellular receptor, Toll-like receptor 4 (TLR-4).

**Methods:**

The effects of nebulized AMP, LPS and AMP administered with LPS on lung function, cellular inflammatory infiltrate and cytokine responses were compared between wildtype mice and mice not expressing TLR-4.

**Results:**

The presence of LPS administered with AMP appeared to drive elevated airway resistance and sensitivity via TLR-4. Augmented TLR4 driven eosinophilia and greater TNF-α responses observed in AMP-LPS treated mice independent of TLR-4 expression, suggests activation of allergic responses by TLR4 and non-TLR4 pathways larger than those induced by LPS administered alone. Treatment with AMP induced macrophage recruitment independent of TLR-4 expression.

**Conclusions:**

These findings suggest AMP-LPS as a stronger stimulus for allergic inflammation in the airways then LPS alone.

**Electronic supplementary material:**

The online version of this article (10.1186/s12931-017-0701-z) contains supplementary material, which is available to authorized users.

## Background

Exposure to ambient air pollution is an adverse health risk to respiratory health, particularly in the young, elderly and those with co-morbidities such as heart disease [1, 2]. In the young, epidemiological and toxicological research studies consistently demonstrate air pollution as a major risk factor in the onset of asthma [3, 4]. This is well illustrated by rising rates of asthma observed in developing countries such as China where expanding industrialization correlates with raised airborne pollution [1]. While in the elderly, long term exposure to particulate matter (PM) has been implicated in developing COPD [1, 2, 5, 6]. Not surprisingly, hospital admission rates for breathing difficulties have been shown to rise during times of raised ambient air pollution concentrations [3]. Despite the risks of air pollution exposure being well accepted, the precise mechanisms leading to the onset of these chronic airway diseases are poorly understood [6–9].

Ambient air pollution, comprised in part by AMP, is a complex mixture of organic compounds, different sized particles and chemicals [10]. The US EPA refers to the inhalable solid phase of ambient air pollution as PM categorized as; coarse (≤ 10 μm), fine (≤ 2.5 μm) and ultrafine (≤ 0.1 μm) [11]. Accumulation mode particles (AMP) straddle the ultrafine particulate (UFP) and fine categories making up the inhalable vapour cloud of PM [12]. AMP’s are largely sourced from engine combustion. Due to their small size these are subject to wind and other climatic conditions which enable dispersal and exposure far from their source of origin [13]. As AMP are small enough to penetrate alveolar spaces and capillary walls, exposure to this particulate size fraction has been shown to result in respiratory disease and exacerbation, with exposure also linked to cardiovascular disease [12, 13].

To date, the majority of air pollution toxicology studies have explored the role of whole ambient mixtures and individual chemical components on respiratory health [7–9]. Due to the number of stimuli within ambient air, identifying the causes and/or interactions responsible for the onset of disease is difficult, as these can trigger a variety of host defence mechanisms when inhaled [14–16]. Oxidative particulates and/or reactive oxygen species generated by particulate phagocytosis have been shown to drive proinflammatory pathways which can cause long-term lung damage and airway disease [17–19]. There is also evidence to suggest Toll like receptor (TLR)-2 and TLR-4 activation in these PM driven inflammatory processes as part of an inflammasome driven response [20–23].

The TLR family are well described pattern recognition receptors that detect characteristic microbial motifs to signal the presence of invading microbial organisms [24]. TLR function forms part of the innate immune system and induce pro-inflammatory cytokine release. These signals alert and activate surrounding tissues and the adaptive immune system [24]. Bacteria are detected by TLR-2 and TLR-4 which recognise components of Gram positive and Gram negative bacterial cell walls known as lipotoeic acid (LTA) and lipopolysaccharide (LPS, also known as endotoxin) respectively [24]. Recognition of either LTA and LPS by TLR’s induces a cascading inflammatory response which can be severe as in the case of sepsis when bacteria are found in blood [25].

Both LTA and LPS form a significant immuno-stimulatory component of ambient air. This has been shown by reduced inflammatory responses in cell cultures treated with ambient PM preparations mixed with polymixin B, a compound binding the Lipid A moiety of LPS [26]. While exposure to LPS has been shown to exacerbate asthma there is conflicting evidence to suggest it also modulates allergic airway responses [27, 28]. The role of TLR-2 and TLR-4 in responses to ambient PM has been further elucidated in alveolar macrophages, a key phagocyte in the lung [29]. However, the overall effects of LPS and PM (including AMP) deposited in the lower airways and the impact of this on lung function and immune modulation has not been fully investigated.

In this study we aimed to delineate the individual and combined effects of LPS and AMP on airway inflammation and potential contribution to airway disease. Due to the well documented inflammatory effects of LPS, we hypothesized that airway inflammation induced by exposure to AMP would be augmented when AMP was co-administrated with LPS. Using a mouse model, the inhaled effects of nebulised AMP, LPS and AMP administered together with LPS on lung function, inflammatory cell infiltrate and cytokine responses in bronchoalveolar lavage and lung parenchymal tissue. To determine the role of TLR-4 in AMP and LPS induced airway inflammation, results were compared between wildtype mice and mice not expressing TLR-4. As PM size fractions contain a mixture of compounds which includes attached LPS [10, 30], an inert fluorescent bead model was used in order to clearly assess the impact of AMP delivered with a known amount of LPS attached.

## Methods

### Animals

We used commercially available fluorescent polystyrene beads as a model for inert AMP (Fluoresbrite™ polychromatic red microspheres, Polysciences Inc., Pennsylvania, USA; herein referred to as AMP) exposure as has been used previously [31]. In this study, mice with a mutated non-functional TLR-4 expression (C3H/HeJ, referred to as TLR4−/−) and mice of the same strain expressing TLR-4 (C3H/HeN, referred to as wildtype, WT) were used in order to assess changes in respiratory mechanics and lung inflammation in response to nebulized treatments of AMP, LPS and a mixed AMP-LPS preparation. TLR4−/− mice have been previously characterized with dysfunctional TLR-4 expression due to a spontaneous proline to histidine point mutation in the TLR-4 signaling sequence [32]. This mouse model is commonly used as a negative control for TLR-4 expression in studies investigating TLR-4 responses [33, 34]. Mice were used at 7–9 weeks of age (Animal Resource Centre, Murdoch, Western Australia) and housed in a controlled environment with a 12 h light to dark cycle with unrestricted access to food and water. All experiments presented were approved by the Telethon Kids Institute’s Animal Ethics Committee (approval reference #128) and carried out in accordance with the recommendations of the Australian code for the care and use of animals for scientific purposes 8th edition (2013).

Ten mice of each strain were grouped to receive the following nebulized treatments: LPS alone (50 μg/ml, *Salmonella typhimurium*, Sigma-Aldrich, St. Louis, Missouri, USA); 0.5 μm polystyrene beads alone (50 μg/ml, equating to approximately 7.26 × 10^10^ particles/ml of AMP; both AMP and LPS (AMP-LPS, 50 μg/mL); or double-distilled water (control). Double distilled water and AMP nebulization preparations contained < 0.015 EU/ml (detection limit) using the Limulus amebocyte lysate (LAL) assay (Sigma-Aldrich, Missouri, USA). Double distilled water induced less airway resistance to methacholine at doses above 3 mg/ml during challenge compared to endotoxin free 0.9% saline, confirming suitability of this as a control for these studies (see Additional file 1: Figure S1) [35]. To best represent short term exposure inducing an inflammatory response, mice were exposed to their allocated treatment at constant flow of 3 ml/min for 30 min at the same time for six consecutive days. Nebulized aerosols were delivered to animals via an UltraNeb™ nebulizer (DeVilbiss, Somerset, Pennsylvania, USA), as described previously [36]. According to DeVilbiss, nebulized droplet size distribution generated ranges from 0.5-3 μm [37]. As a solution of 0.5 μm polychromatic spheres were used, the overall nebulization range was deemed to fit the accumulation mode particle size range (0.1–2.5 μm) for the purposes of this study.

### Lung function measurements

Lung function was assessed using a modification of the low frequency, forced oscillation technique (LFOT)*.* Mice were initially anaesthetized with an intraperitoneal injection of a solution containing xylazine (2 mg/ml, Troy Laboratories, NSW, Australia) and ketamine (40 mg/ml, Troy Laboratories, NSW, Australia) at a dose of 0.01 mg/g. Mice were then tracheotomized and a 10 mm section of polyethylene tubing (1.27 mm OD, 0.86 mm ID) inserted into the trachea. Mice were ventilated at 450 breaths/min with a tidal volume of 8 ml/kg and a positive end expiratory pressure (PEEP) of 2 cm H_2_O using a computer-controlled ventilator (flexiVent, SCIREQ Inc., Montreal, Canada). This system was used for ventilation and measurement of respiratory mechanics as previously described [38, 39].

Before commencing lung function measurements, mouse lung volume history was standardized using 5 deep inflations to total lung capacity. Respiratory impedance (Zrs) was measured using an oscillatory signal containing 19 frequencies ranging from 0.25 to 19.625 Hz during pauses in ventilation. Zrs was partitioned into components representing the mechanical properties of the airways and lung tissue parenchyma using a four parameter model with constant phase tissue impedance [38, 39]. Partitioning of Zrs in this way allows calculation of parameters representing airway resistance, tissue damping and tissue elastance [40, 41].

### Methacholine challenge

Following measurement of baseline Zrs, mice were exposed to a saline aerosol for 90s (Ultraneb™ 99, Devilbiss, Somerset, Pennsylvania, USA). Five measurements of Zrs were then obtained, averaged and used as the control measurements for MCh challenge. The aerosol procedure was repeated with half log incremental doses of MCh from 0.1 to 30 mg/ml. Measurements of Zrs were recorded every minute for 5 min after each MCh aerosol and the maximum response calculated. From these, data dose response curves for airway resistance (Raw) were constructed. Sensitivity to MCh was determined by calculating the MCh dose required to produce a 200% increase in Raw in response to the saline challenge at 30 mg/ml using interpolation [41]. Maximum responses in Raw and airway sensitivity were used to compare lung function responses between groups.

### Inflammatory cell counts

Five additional animals per group were anaesthetised and tracheotomised for bronchoalveolar lavage (BAL) used for inflammatory cell infiltrate and inflammatory cytokines analysis as previously described [40]. Briefly, BAL fluid was collected by slowly infusing and withdrawing a 1 ml aliquot of 0.9% saline from the lung three times. The resulting fluid was centrifuged at 2000 rpm for 4 min. Supernatant was collected and stored at −80 °C for later analysis. The cell pellet was resuspended in saline and a portion stained with trypan blue to determine viability and total cell count (TCC). The remaining portion was centrifuged onto slides and stained with Leishman’s (Sigma-Aldrich, St Louis, Missouri, USA) to obtain differential cell counts.

### Inflammatory cytokine responses

#### Bronchoalveolar lavage (BAL)

Analysis of BAL for the presence of secreted pro-inflammatory cytokines known to be secreted in response to LPS and PM [42]. Interleukin (IL)-6, Interferon (IFN)-γ, and Tumor necrosis factor (TNF)-α was completed using a cytokine bead array assay (BD Biosciences California, USA) as per manufacturers’ instructions, with a detection range of 20-5000 pg/ml for all cytokines within the array. These measurements were completed in BAL from five mice of each mouse strain for each treatment using an optimized sample dilution factor.

#### Lung parenchyma

Soluble protein was extracted from a single mouse lung lobe to gain a measure of subepithelial pro-inflammatory responses indicative of airway remodelling and developing chronic airway inflammation. For this reason expression of immune-regulatory, IL-10 and pro-fibrotic cytokine, IL-13, were examined using ELISA (R&D Systems, Abdington, UK; detection ranges: IL-10, 31.2-2000 pg/ml and IL-13, 62.5-4000 pg/ml) using optimised sample dilution factors. Specifically, IL-10 was chosen as it is secreted in response to LPS and is thought to protect the lung against lung injury by reducing the production of proinflammatory cytokines, chemokines and transcription factors implicated in airway remodelling. Whereas IL-13 is known to be involved in subepithelial fibrosis related to the onset of asthma and COPD. Data was calculated and normalized to 100 μg of total soluble protein as measured using the Pierce BCA assay (Thermo-Pierce, Rockford, USA) for comparative purposes.

### Statistical analysis

SPSS Levene’s test was used to test for equal variance across all the groups of data compared. Following verification, an independent t-test was then used to determine statistically significant differences between (a) controls and treatment groups and (b) single treatments between WT and TLR4−/− mice. Due to differences in baseline lung function (see results Table 1), responses to MCh were expressed as a percentage of baseline with graphs are shown as mean ± SEM. Due to the range of data, cytokine responses are presented as box and whisper plots depicting interquartile range and 2.5 and 97.5 percentiles with medians. A minimum of 4 biological replicates and *p* values < 0.05 considered significantly different were used for all data sets.

**Table 1:** Baseline lung function for mice studies completed in control and treated wildtype (WT) and TLR4 (TLR4 −/−) mutant mice. Baseline lung function measurements taken before methacholine challenge were not significantly different between treatments in WT and TLR4−/− mice. Greater Raw values were measured for all TLR4−/− mice compared to WT mice of the same treatment, indicating that overall, this mouse strain had more sensitive airways

*Treatment*	*Control*		*AMP*		*LPS*		*AMP-LPS*	
*Mouse strain*	*WT*	*TLR4−/−*	*WT*	*TLR4−/−*	*WT*	*TLR4−/−*	*WT*	*TLR4−/−*
Raw (hPa.s.ml^−1^)	0.35	0.45*	0.34	0.46*	0.34	0.40*	0.34	0.45*
	(0.01)	(0.02)	(0.01)	(0.02)	(0.01)	(0.03)	(0.01)	(0.02)

*p < 0.05 between WT and TLR4−/−,()indicates SD

## Results

### Lung function

Baseline lung function responses were recorded for each animal prior to saline and methacholine (MCh) challenge (Table 1). Baseline airway resistance (Raw) values were elevated in all TLR4−/− mice compared to wildtype (WT) mice of the same treatment (PM *p* = 0.04, LPS *p* = 0.03, PM-LPS *p* = 0.035), including control mice (*p* = 0.035); indicating this mouse strain had more sensitive airways overall. However, baseline Raw did not vary considerably between mice of the same strain treated with AMP, LPS or AMP-LPS nebulisations (Table 1).

**Fig. 1:**
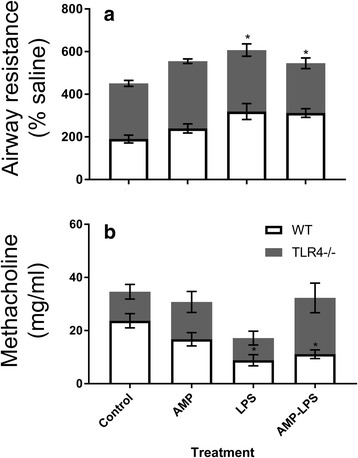
Airway resistance (**a**) and sensitivity to methacholine at 30 mg/ml (**b**) for wildtype (WT) and mice not expressing TLR4 (TLR4−/−) for each treatment. Airway resistance (Raw) values for the largest methacholine challenge (MCh, 30 mg/ml) are presented as a percentage of the initial saline challenge given to each animal given prior to commencing MCh challenges (**a**). Raw was augmented in WT mice treated with nebulized LPS and AMP-LPS only. Airway sensitivity was calculated by interpolating the amount of MCh needed to cause a doubling of baseline responses (**b**). WT mice treated with LPS and AMP-LPS required significantly less MCh than that needed for control mice, indicating more sensitive airways due to LPS and AMP-LPS treatments in the presence to TLR-4 (*p < 0.05 compared to controls). No significant differences were observed in airway resistance or sensitivity between treatments of LPS or AMP-LPS within each mice strain (*p* > 0.05)

At 30 mg/ml MCh, Raw was significantly augmented in WT mice treated with nebulized LPS or AMP-LPS compared to control mice (*p* = 0.04 and 0.03 respectively, Fig. 1a). Sensitivity to MCh as determined by interpolation, was significantly less in WT mice treated with LPS (*p* = 0.008) and AMP-LPS (*p* = 0.017) compared to controls. This was not observed in LPS and AMP-LPS treated TLR4−/− mice, indicating more sensitive airways in response to LPS and AMP-LPS treatments in the presence of TLR-4 (Fig. 1b). Responses for all other doses of MCh used for challenge can be found in Additional file 2: Figure S2.

### Cellular responses measured in bronchoalveolar lavage

**Fig. 2:**
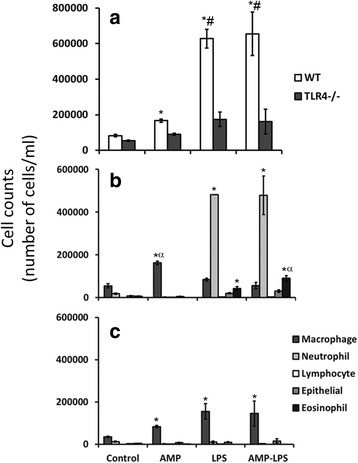
Bronchoalveolar lavage (BAL) total (**a**) and differential cell counts (**b, c**) for wildtype (WT) and mice not expressing TLR4 (TLR4−/−) for each treatment. Elevated total cell counts in BAL were observed in WT mice treated with AMP, LPS and AMP-LPS compared to WT control mice. Cell counts in WT mice treated with LPS and AMP-LPS were also greater than similarly treated TLR4−/− mice. Neutrophils were the predominant cell type in LPS and AMP-LPS treated WT mice. Macrophages dominated counts in AMP treated WT and TLR4−/− mice, as well as LPS and AMP-LPS treated TLR4−/− mice compared to respective controls. Greater eosinophil numbers were observed in WT mice treated with LPS and AMP-LPS; for which numbers were greater in AMP-LPS treated mice. Lymphocyte and epithelial cell numbers were not significantly different between controls and any of the treatments given for either strain (*p < 0.05 between treatment and control; # = *p* < 0.05 between strains for the same treatment; α = *p* < 0.05 between treatment compared to all other treatments for the same strain)

Bronchoalveolar lavage (BAL) inflammatory total cell counts were larger in WT mice treated with AMP, LPS (*p* < 0.001) and AMP-LPS (*p* < 0.001) compared to WT control mice. Total cell counts in WT mice treated with LPS (*p* < 0.001) and AMP-LPS (*p* < 0.001) were greater than similarly treated TLR4−/− mice (Fig. 2a). Neutrophils were the predominant cell type in LPS and AMP-LPS treated WT mice compared to control and AMP treated mice (*p* < 0.001) **(**Fig. 2b**).** In contrast, neutrophils were barely detectable in TLR4 −/− mice irrespective of exposure. Macrophages were the dominant cell type in mice treated with AMP irrespective of strain (*p* = 0.01 for both strains) and in TLR4−/− mice treated with LPS (*p* = 0.007) and AMP-LPS (*p* = 0.04) compared to respective controls (Fig. 2c**)**. Eosinophil numbers were greater in WT mice treated with LPS (*p* = 0.006) and AMP-LPS (*p* < 0.001); for which numbers were larger in AMP-LPS treated mice (*p* = 0.024) compared to those treated with LPS. Lymphocyte and epithelial cell numbers were not significantly different between controls and any of the treatments given for either strain. Other cytokine responses measured using the commercial kit can be found in Additional file 3: Figure S3.

### Inflammatory cytokine responses

#### Bronchoalveolar lavage

**Fig. 3:**
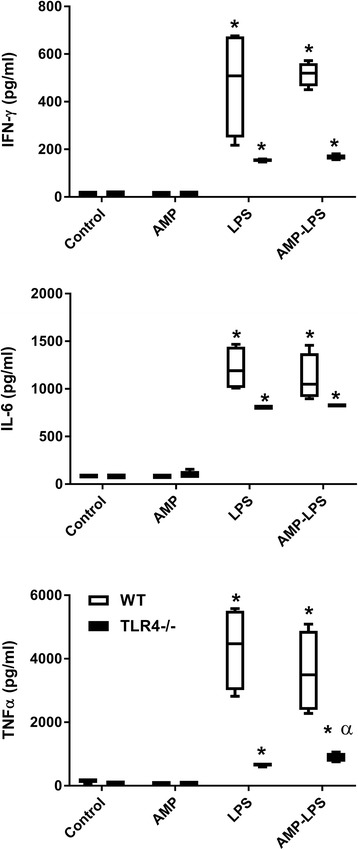
Cytokine responses measured in bronchoalveolar lavage collected from treated wildtype (WT) (□) and mice not expressing TLR4 (TLR4−/−) (■). Significantly elevated IFN-γ, IL-6 and TNF-α was observed in WT and TLR4−/− mice treated with LPS and AMP-LPS, with these results being greater in WT mice. Only AMP-LPS treated TLR4−/− mice had significantly more TNF-α compared to LPS treated TLR4−/− mice. The amount of these cytokines was not significantly different in AMP treated mice compared to control mice for both strains (* indicates *p* < 0.05 compared to controls, α = *p* < 0.05 single treatment compared to all other treatments for the same strain)

Significantly increased levels of IFN-γ, IL-6 and TNF-α were observed in wildtype and TLR4−/− mice treated with LPS (Wildtype: IFN-γ *p* = 0.02; IL-6 *p* = 0.002; TNF-α *p* = 0.001, TLR4−/−: IFN-γ *p* < 0.001; IL-6 *p* < 0.001; TNF-α *p* < 0.001) and AMP- LPS (Wildtype: IFN-γ *p* < 0.001, IL-6 *p* < 0.001, TNF-α *p* = 0.001, TLR4−/−: IFN-γ *p* < 0.001; IL-6 *p* < 0.001; TNF-α *p* < 0.001), with greater amount of cytokine in wildtype mice for these treatments (LPS *p* = 0.028 and APM-LPS *p* < 0.001) (Fig. 3). Only AMP-LPS treated TLR4−/− mice had significantly more TNF-α compared to LPS treated TLR4−/− mice (*p* = 0.032). The amounts of these cytokines were not significantly different in AMP treated mice compared to control mice for both strains.

#### Lung parenchyma

**Fig. 4:**
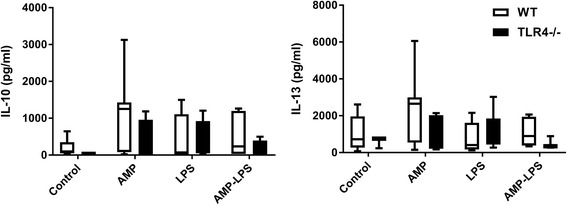
Cytokines measured in lung parenchymal tissue from wildtype (WT) (□) and mice not expressing TLR4 (TLR4−/−) (■) treated with AMP, LPS and AMP-LPS. There were no observed differences in IL-10 and IL-13 protein expression measured from whole mouse lung lobe analysed by ELISA for between mouse strains for any treatment or for any treatment compared to control non-treated mice (*p* > 0.05). Results were normalised to 100 μg of total soluble protein for comparative purposes (*n* = minimum of 4 for each group)

Soluble protein was extracted from one whole mouse lung lobe and analysed by ELISA for IL-10 and IL-13 expression showed no differences in these cytokines for any treatment in WT or TLR4−/− mice compared to controls (*p* > 0.05 for all; Fig. 4). Similarly, there were no differences observed between WT and TLR4−/− mice for any of the individual inhaled treatments administered (*p* > 0.05).

## Discussion

The results of this study clearly demonstrate that the inflammatory effects of inhaled particulate matter are heavily influenced by the presence of LPS. Airway resistance and sensitivity were shown to correlate inflammatory cytokine responses to inhaled LPS and AMP-LPS measured in bronchoavleolar lavage. While these responses were more pronounced when signalled by TLR-4, inflammation was also observed in TLR-4 knock-out mice indicating other LPS recognition pathways. A larger TNF-α response observed in TLR-4 knockout mice treated with AMP-LPS compared to LPS alone, suggest alternate recognition or a divergent signalling pathway for this treatment combination. As there were no changes observed in IL-10 or IL-13 expression measured in lung parenchymal tissue, this suggests the inhaled preparations used in this study did not have an effect on airway tissue remodelling. Interestingly, elevated macrophage numbers observed in mice treated with inhaled latex beads alone, used as the model for AMP in this study, was not mimicked by increased inflammatory cytokine levels or augmented airway responses when compared to control non-treated mice.

Raised ambient PM levels are shown to be directly correlated to asthma admissions in health care centres, with long term exposure linked to the onset of lung cancer and COPD [2]. In this study, we did not find any significant change in lung function resulting from AMP exposure. However, augmented airway resistance and airway sensitivity responses to methacholine were observed in wildtype mice exposed to LPS and AMP-LPS. As LPS is found ubiquitously in the environment our data suggests LPS attached to inhalable AMP induces changes in lung function rather than AMP alone. As AMP exposure is linked to the onset of chronic diseases such as COPD, asthma and even cardiovascular disease a longer study period may be more suitable. This would allow tracking of slow onset of symptoms which underlie these diseases in response to ongoing long-term exposure to inhaled AMP.

Neutrophilic inflammation present in wildtype mice treated with LPS and AMP-LPS compared to TLR4−/− mice indicates this response was driven by the presence of TLR-4 driven by the presence of LPS. In the absence of TLR-4, cellular inflammation to LPS and AMP-LPS was dominated by the presence of macrophages. AMP alone also induced increased macrophage infiltration compared to control mice, however this was observed irrespective of TLR-4 expression. Elevated macrophage numbers suggests either strengthened recruitment to the lung to clear inhaled particles, or impaired clearance, a hallmark of alveolar macrophages overloaded with phagocytosed particles [43–48]. On the other hand, larger neutrophil numbers in response to particle inhalation have been shown to correlate the onset of cancer tumors, an observation which dissipates when particle deposition shifts from the alveolar space to lung interstitium [49]. Airway deposition of AMP particles was not characterised in this study; however, these observations clearly demonstrate a greater number of macrophages with unchanged neutrophil numbers compared to non-treated control mice. Therefore, these findings suggest an interstitial lung deposition of AMP with induced inflammatory responses independent of TLR-4 expression for the first time. Interestingly, eosinophil numbers were significantly higher in wildtype mice treated with AMP-LPS compared to LPS. Indeed distinct TLR-4 driven cellular compartments have been shown to activate neutrophilic and eosinophilic responses in response to different allergens [50], which may explain the larger eosinophil responses observed in wildtype mice treated with AMP-LPS compared to LPS alone. As eosinophilia is closely associated with the onset of asthma and allergy [50, 51], further investigation of this finding may elucidate the cellular mechanisms underlying allergic airway disease caused by exposure to particulates.

Of those treated wildtype mice, LPS or AMP-LPS induced the largest inflammatory cytokine responses measured in BAL. Augmented responses to LPS and AMP-LPS were also observed in TLR4−/− mice, illustrating proinflammatory signalling mechanisms other than TLR-4 activated by LPS. Furthermore, TNF-α levels were significantly greater in BAL of AMP-LPS treated TLR4−/− mice compared to LPS treated mice, suggesting this combination was signalled by yet another mechanism. As we found evidence for LPS being attached to AMP, elevated TNF- α levels may have been induced by alternate receptors for LPS (such as scavenger receptors), or endocytosed resulting in recognition by intracellular pattern recognition receptors for LPS; including nucleotide-binding oligomerization domain (NOD) receptors contained in cellular inflammasomes [51]. Indeed, Shi et al. have shown binding of LPS by caspase 11 is critical for activation of this intracellular process [51]. Augmented TNF-α responses have been shown in the presence of eosinophilia [52, 53]. Thereby the combination of elevated non-TLR4 driven TNF-α and TLR-4 driven eosinophilia observed in AMP-LPS treated mice suggest AMP-LPS is a stronger stimulus for allergic inflammation in the airways than LPS alone. Despite a larger number of macrophages observed in AMP treated mice, these did not display inflammatory cytokine responses that were significantly different to those measured in non-treated control mice.

Interestingly, IL-10 or IL-13 measured in the lung parenchyma remained unchanged in response to all inhaled treatments for wildtype and TLR4−/− mice. This is surprising given the long-standing relationships between AMP exposure and airway disease development characterised by airway remodelling co-ordinated by these cytokines [54, 55]. Within the context of this study, this novel finding suggests TLR-4 driven inflammatory responses activated by LPS recognition appear to be predominantly secreted (BAL). However, as long term-low grade inflammation activity can go undetected in subepithelial tissues for long periods [55], a larger study period may elucidate mechanisms pertinent to slow onset airway disease attributable to AMP exposure such as COPD and related cardiovascular disease [56]. Importantly, IL-13 responses are associated with allergen associated airway disease such as asthma [57]. Elevated TNF-α responses measured in BAL to LPS suggests modulation of this response by type-2 inflammatory cytokines such as IL-4 or IL-5. Although closely affiliated with IL-4, IL-13 responses observed in lung parenchyma did not correlate LPS induced TNF-α responses in BAL [58]. Therefore, findings from a longer study period which include analysis of IL-4 or IL-5 may be valuable to our overall understanding of immune-modulated airway disease in response to allergic stimuli carried in inhaled air such as LPS, which has remained elusive to date [9].

## Conclusions

In conclusion, we have shown the presence of LPS in AMP preparations has an influential impact on induced airway and inflammatory BAL responses in the lung which are augmented by the presence of TLR-4. Importantly, dominant macrophage responses observed in BAL from AMP treated mice over all other treatments, suggest interstitial lung deposition, triggered regardless of TLR-4 expression for the first time. Despite this, inflammatory cytokine responses were not observed in the lung parenchymal tissues in response to any treatment, suggesting a longer study period may be needed to observe pro-fibrotic changes that underlie airway disease caused by long-term AMP inhalation. Interestingly, when AMP was attached to LPS larger TNF-α responses independent of TLR-4 expression were observed in BAL suggesting activation of allergic responses by non-TLR4 pathways. If augmented by TLR-4 driven eosinophilia as observed in AMP-LPS treated mice, these findings suggest AMP-LPS as a stronger stimulus for allergic inflammation in the airways over LPS alone. Taken together, these results demonstrate divergent response pathways in the lung to AMP and LPS, with larger allergy affects observed in AMP-LPS which have not been shown before. Therefore, these findings contribute novel information to the field investigating the onset of allergic and non-allergic airway disease, such as asthma and COPD, as a result of PM exposure and warrants further investigation.

## Additional files

Additional file 1: Figure S1. Airway resistance in TLR4−/− mice treated with double distilled water (ddH_2_O) and saline. Saline responses were significantly greater for methacholine challenges larger than 3 mg/ml (* *p* < 0.05). (TIFF 356 kb)

Additional file 2: Figure S2. Airway resistance in wildtype (WT) and TLR4−/− mice for all treatment groups across for all methacholine challenges used. Raw was significantly greater in WT mice treated with LPS and AMP-LPS compared to control mice at 30 mg/ml MCh (*p < 0.05). (TIFF 1196 kb)

Additional file 3: Figure S3. Additional cytokines measured in bronchoalveolar lavage (BAL) and lung parenchyma. MCP-1 was measured in BAL using cytokine bead array assay (20-5000 pg/ml detection range) and IL-8 in lung parenchyma using ELISA (15.6-1000 pg/ml detection range) using optimised sample dilution factors. No significant difference with treatment was observed for these cytokines. (TIFF 558 kb)
