# Supplementary figures and images for: Accumulation mode particles and LPS exposure induce TLR-4 dependent and independent inflammatory responses in the lung
Source: Respir Res. 2018 Jan 22;19:15. doi: 10.1186/s12931-017-0701-z (PMC5778683; doi:10.1186/s12931-017-0701-z)

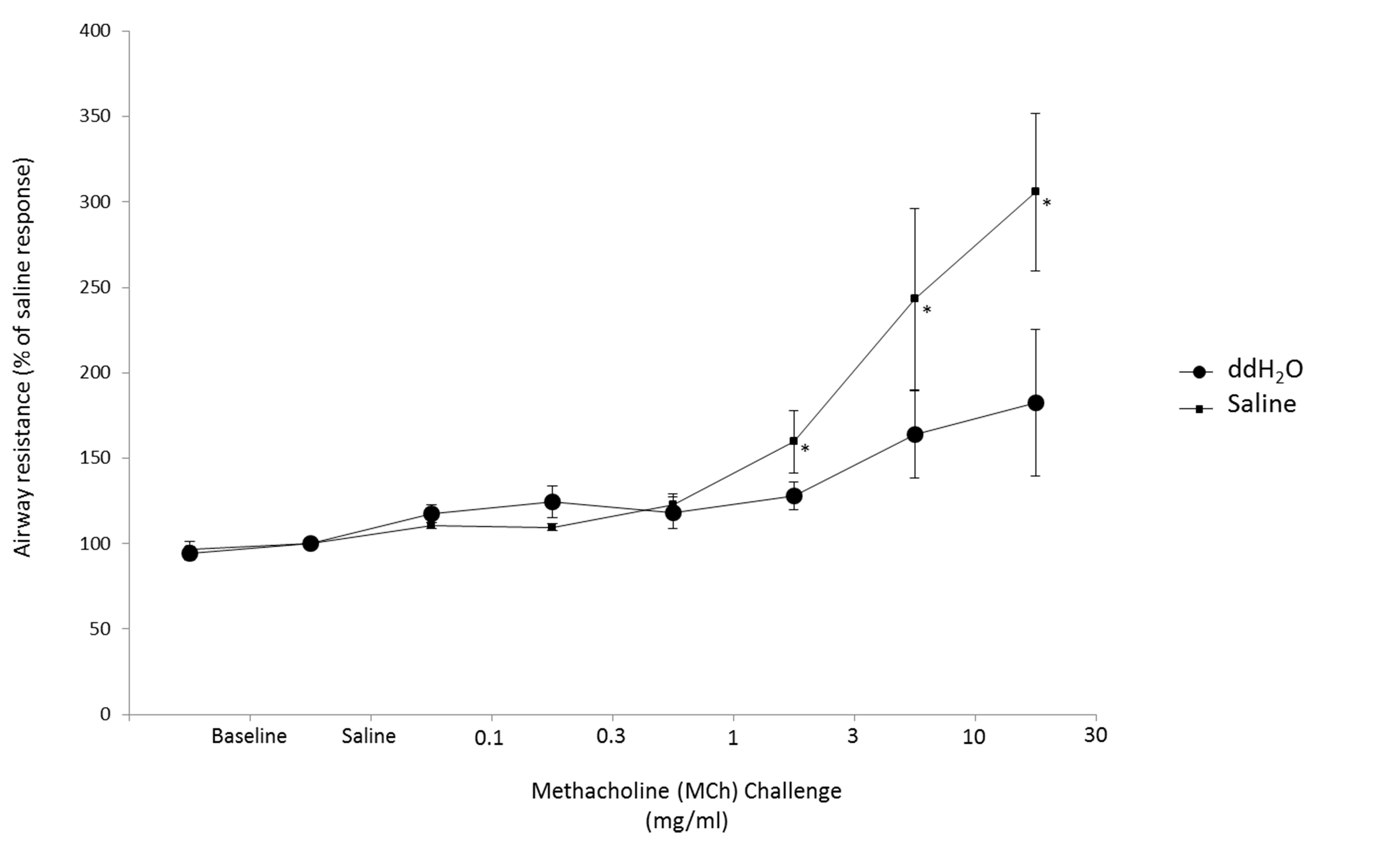

Supplement: Supplementary file 1 — Airway resistance in TLR4−/− mice treated with double distilled water (ddH2O) and saline. Saline responses were significantly greater for methacholine challenges larger than 3 mg/ml (* p < 0.05). (TIFF 356 kb) [file 12931_2017_701_MOESM1_ESM.tif]

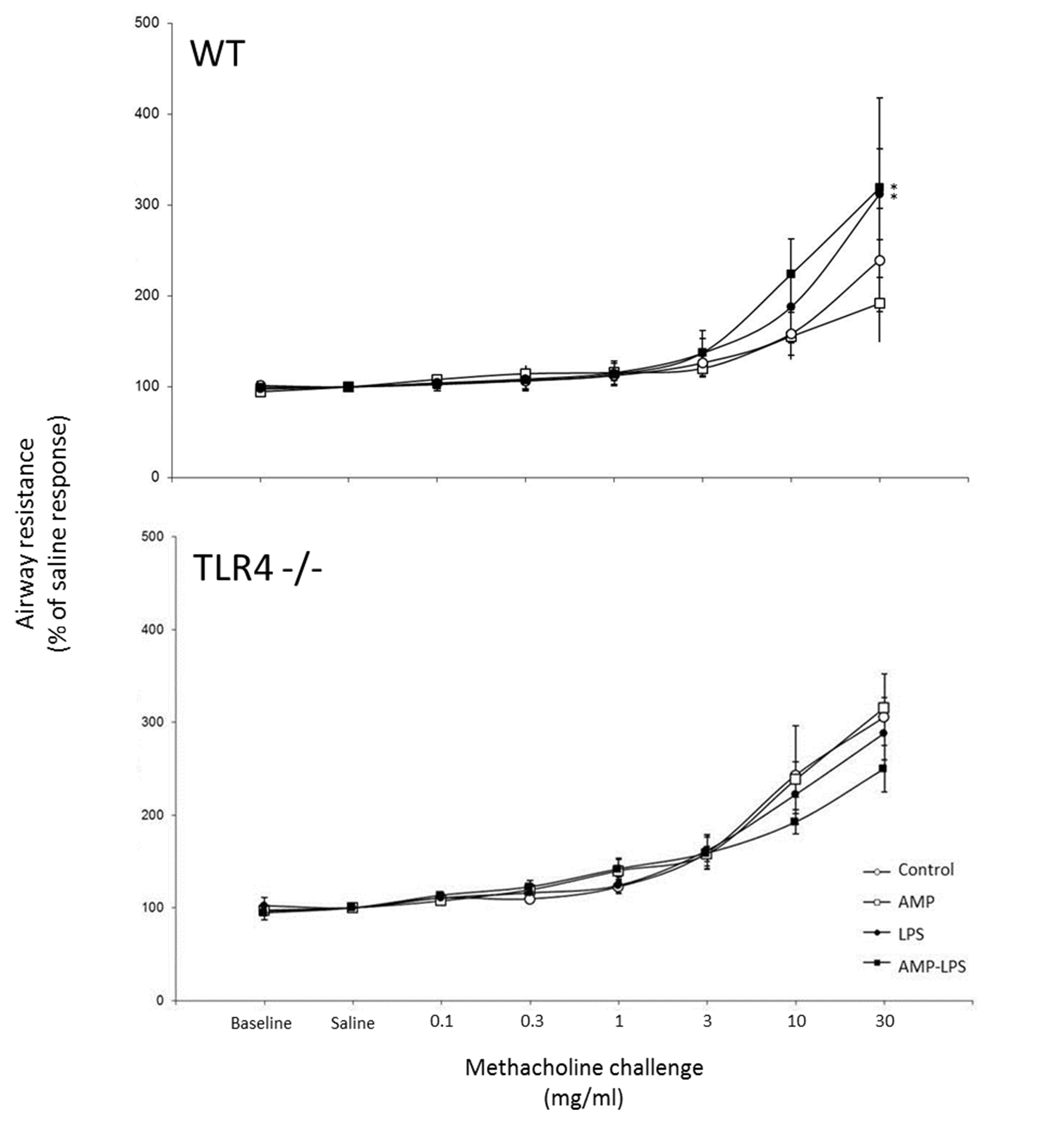

Supplement: Supplementary file 2 — Airway resistance in wildtype (WT) and TLR4−/− mice for all treatment groups across for all methacholine challenges used. Raw was significantly greater in WT mice treated with LPS and AMP-LPS compared to control mice at 30 mg/ml MCh (*p < 0.05). (TIFF 1196 kb) [file 12931_2017_701_MOESM2_ESM.tif]

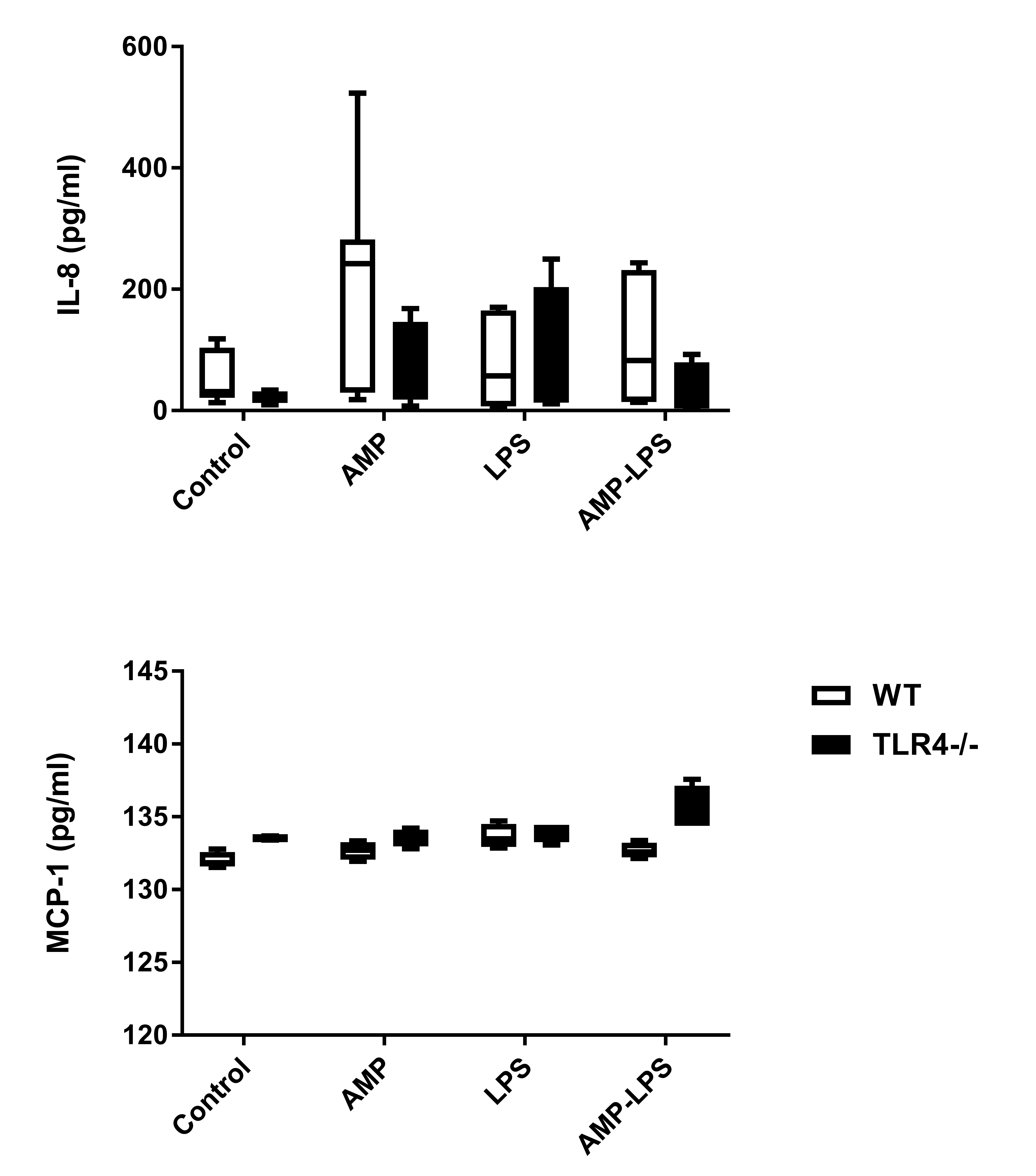

Supplement: Supplementary file 3 — Additional cytokines measured in bronchoalveolar lavage (BAL) and lung parenchyma. MCP-1 was measured in BAL using cytokine bead array assay (20-5000 pg/ml detection range) and IL-8 in lung parenchyma using ELISA (15.6-1000 pg/ml detection range) using optimised sample dilution factors. No significant difference with treatment was observed for these cytokines. (TIFF 558 kb) [file 12931_2017_701_MOESM3_ESM.tif]
